# Frequency of Mutations Within P‐Glycoprotein and Beta‐Tubulin Associated With Ivermectin Treatment in *Onchocerca volvulus* Adult Female Worm Populations in Some Onchocerciasis Endemic Communities in Ghana

**DOI:** 10.1155/japr/3136302

**Published:** 2026-10-07

**Authors:** Rufus Akinkagle, Millicent Opoku, Millicent Selassie Afatodzie, Joseph Harold Nyarko Osei, Sampson Otoo, Sarah-Sally Mawunyo Dogbe, Abena Akyeamaa Nyarko, Martin N. Donkor, Mawuli K. Azameti, Iddrisu Fuseini, Sellase Pi-Bansa, Daniel Adjei Boakye, Millicent Captain-Esoah, Kwadwo Kyereme Frempong

**Affiliations:** ^1^ Department of Applied Biology School of Environment and Life Sciences University of Technology and Applied Sciences Navrongo Ghana hct.edu.om; ^2^ Department of Parasite and Vector Biology Noguchi Memorial Institute for Medical Research College of Health Sciences University of Ghana Legon Ghana ug.edu.gh; ^3^ Department of Ecological Plant and Animal Sciences School of Agriculture Biomedicine and Environment La Trobe University Bundoora Australia latrobe.edu.au; ^4^ Biomedical and Public Health Research Unit Water Research Institute Council for Scientific and Industrial Research Accra Ghana csir.org.gh; ^5^ Department of Biochemistry and Forensic Sciences School of Chemical and Biochemical Sciences University of Technology and Applied Sciences Navrongo Ghana hct.edu.om; ^6^ The END Fund 2 Park Avenue New York USA

**Keywords:** adult female worm, beta-tubulin, frequencies of mutations, Ghana, ivermectin, *Onchocerca volvulus*, onchocerciasis, p-glycoprotein

## Abstract

**Background:**

Ivermectin is the drug of choice for the control of onchocerciasis because of its potent embryostatic and microfilaricidal effect. However, with long periods of mass drug administration, the effect of ivermectin on the *Onchocerca volvulus* worms is waning. These suboptimal responses have been linked to genes such as p‐glycoprotein (*P-gp*) and beta‐tubulin (*B-tub*). The purpose of this study was to define the proportion of adult female *O. volvulus* from some endemic communities in Ghana that have mutations in these genes.

**Methods:**

*O. volvulus* adult female worms were obtained from 33 participants in four endemic communities. Genomic DNA was isolated from worms and evaluated utilising polymerase chain reaction‐restriction fragment length polymorphism (PCR‐RFLP) using *Dde*I and *Alu*I restriction enzymes to identify genetic mutations within *P-gp* and *B-tub* genes. The wild‐type and mutant forms of the genes were identified and associations between the frequencies of the mutations and ivermectin treatment were determined.

**Results:**

A total of 45 adult female worms were obtained from 33 participants, and about 40% of these worms were reproductively active. High frequencies of mutant genes observed in parasite population (91.1%) for the *B-tub* gene and all the samples (100%) for the *P-gp* gene. Irrespective of these high frequencies in mutation, a Fisher’s Exact Test (*p* = 0.621) and Mann–Whitney *U* test (*p* = 1.000) showed no significant relation between mutations and community rounds of ivermectin or individual participant’s treatment.

**Conclusion:**

This study shows a high frequency of mutations in both *P-gp* and *B-tub* genes in *O. volvulus* isolates, indicating the possible involvement of genetic changes in suboptimal response of the disease to ivermectin in parasite population. Continuous molecular surveillance to monitor emerging resistance is important to guide future strategies towards elimination.

## 1. Introduction

Human onchocerciasis, commonly known as river blindness, is a debilitating parasitic disease caused by the filarial worm *Onchocerca volvulus* and transmitted by blackflies of the genus *Simulium* through repeated infectious bites [1]. The adult worm of the parasite resides in the subcutaneous nodules, while the microfilariae migrate into the epidermis of the skin and ocular tissues, causing severe itching, skin depigmentation and blindness. The disease remains a major public health problem in sub‐Saharan Africa, where more than 99% of global cases occur [1]. As one of the important neglected tropical diseases, onchocerciasis persists, especially in sub‐Saharan Africa, notwithstanding the years of community‐directed treatment with ivermectin (CDTI) in endemic countries [2–5].

In Ghana, onchocerciasis is endemic in 15/16 regions across different ecological zones, particularly in the Volta, Oti, Northern, Western North and Bono regions. Ghana was one of the focal countries in the original Onchocerciasis Control Programme (OCP, 1974–2002) and the subsequent African Programme for Onchocerciasis Control (APOC, 1995–2015). Ivermectin was introduced in Ghana in 1987 with trials beginning in Asubende [6], following its licensing for use in humans as a public health control strategy. Since 1995, the main strategy for control and now elimination has been mass drug administration (MDA) with ivermectin (Mectizan), distributed annually or biannually through CDTI programmes [7, 8].

Ivermectin is a macrocyclic lactone that targets ligand‐gated chloride ion channels in the parasite, particularly the glutamate‐gated chloride channels, leading to paralysis and death of *O. volvulus* microfilariae [9, 10]. However, it does not kill the adult worms at the standard dose (150–200 *μ*g/kg), but rather temporarily sterilises the adult female worms, suppressing microfilariae production for several months. These antimicrofilarial and embryostatic properties of the drug have contributed greatly to curbing the prevalence of the disease [11]. When the drug is given repeatedly in MDA campaigns, it can drastically reduce microfilarial loads, transmission potential and ultimately infection prevalence [12, 13]. Adult female *O. volvulus* may live up to 10–14 years, and the effectiveness of ivermectin depends on maintaining repeated treatments at high coverage for the entire reproductive life span of the worm [8, 14, 15]. Over the years, there have been reported concerns of persistent microfilaridermia in some communities despite long‐term treatment, attributable to suboptimal responses of the adult female worm to the drug [2–5]. This implies that the recovery of the reproductive ability by adult female worm after treatment, as well as the release of the microfilariae, is now shortened.

Studies in Ghana and other West African countries have reported slower‐than‐expected declines in microfilarial prevalence and intensity after many years of MDA with ivermectin [2–5]. In some communities in the Bono and Oti Regions in Ghana, individuals exhibited repopulation of skin microfilariae within 3–6 months post‐treatment—much faster than the typical 9–12 months seen in naive populations [2, 16]. These ‘suboptimal responders’ also exhibited a reduced anti‐fecundity effect of ivermectin on adult female worms. Such phenotypic evidence suggests potential selection for drug‐tolerant worm populations due to repeated MDA pressure [17]. Understanding the molecular mechanisms and genetic markers associated with such responses is critical for early detection and containment of potential ivermectin resistance.

Drug resistance in nematodes, including *O. volvulus*, can arise from mutations in genes coding for drug targets, drug transporters or detoxification systems. Two genes of major interest are beta‐tubulin (*B-tub*) and p‐glycoprotein (*P-gp*), which have been strongly associated with ivermectin treatment [18, 19]. *B-tub* protein forms part of the microtubule structure essential for cell division and intracellular transport [20]. Mutations in *B-tub* genes are well known to mediate benzimidazole resistance in veterinary nematodes such as *Haemonchus contortus* and *Trichostrongylus colubriformis* [21]. In *O. volvulus*, selection pressure from ivermectin exposure has been associated with single nucleotide polymorphisms (SNPs) in the *B-tub* gene [19]. These were significantly more frequent in worms from ‘poor response’ communities. These mutations were hypothesised to be linked to altered gene expression or structural stability that may influence ivermectin susceptibility. *P-gp* belongs to the ATP‐binding cassette (ABC) transporter family and acts as a drug efflux pump, reducing intracellular accumulation of xenobiotics. In nematodes, overexpression or mutation of *P-gp* has been linked to macrocyclic lactone resistance [22], while genetic variation within the *P-gp*‐like gene has been reported to differ between ivermectin‐treated and untreated *O. volvulus* populations in Ghana [23].

Despite previous molecular studies, there remain significant gaps in our understanding of how widespread these mutations are currently across adult worm populations in endemic communities. Data on the frequencies of mutations in the adult worm population is very scanty or outdated in Ghana. Data on the frequencies of drug exposure within the adult female worm are relevant for making decisions, especially as the focus has shifted from onchocerciasis control to elimination. Information on the frequencies and distribution of exposure of ivermectin would be crucial in informing the selection of alternative drugs for the use in endemic settings with persistent transmission. Additionally, MDA with ivermectin in Ghana has been ongoing for over three decades, but there have been reports of emerging suboptimal response of the parasite to the drug in Ghana and other endemic countries [2, 16, 24, 25]. Genetic mutations in *O. volvulus*, specifically *P-gp* and *B-tub* genes, are major contributors to reduced sensitivity of the parasite to the drug [18, 19].

We identified frequencies of mutations within the *P-gp* and *B-tub* genes in adult female *O. volvulus* in some communities from the Oti and Northern regions of Ghana. Additionally, the study determined the association between genetic mutations and community rounds of ivermectin treatment or individual participant’s treatment frequencies. The mutations were mapped to provide a visual geographic distribution. We have provided data to support indication of possible selection pressure and emerging tolerance in the adult worm population for molecular surveillance towards onchocerciasis elimination in Ghana.

## 2. Materials and Methods

### 2.1. Ethics Statement

The ethical approval for this study was sought from the Ghana Health Service Ethics Review Committee (GHS‐ERC:017/02/24).

### 2.2. Study Site

The study was conducted in the Oti and Northern regions of Ghana. Three communities (two adjacent communities: Abunyanya 1 and Abunyanya 2; and Koni) are located in the Nkwanta North District of the Oti region while one community (Wiae) is located in the Kpandai District of the Northern region (Figure 1). Nkwanta North District shares borders with the Republic of Togo, Nanumba South District, Kpandai District and Nkwanta South District. According to the 2021 population and housing census, the Nkwanta North District had a population of 126,096, with 62,622 males (49.7%) and 63,474 females (50.3%), with Kpassa as the district capital [26]. The district has 122 communities, and it lies in a tropical climate zone. It has natural vegetation of transitional savannah woodland, with ethnic groupings such as Konkombas, Basares, Ewes and Akans found in the district.

**Figure 1:**
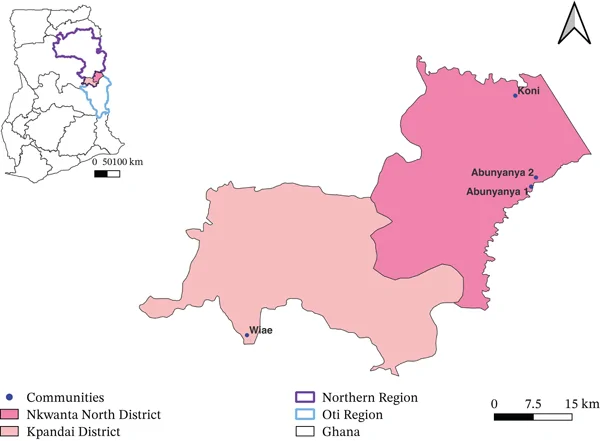
Map showing selected communities and districts where sampling was done within the Northern and Oti regions of Ghana. The base layer for this map was accessed from the ‘Simplemaps—Interactive Maps & Data’ website [https://simplemaps.com/gis/country/gh]. License: Creative Commons Attribution 4.0. The software for drawing this map is ArcGIS v10.4.1 [http://www.esri.com]. The terms of use/license information for the base layer image or shapefile is [https://data.humdata.org/about/license/legacy_hrinfo].

The Kpandai District shares borders with Krachi West, Nkwanta North, Pru, East Gonja and Nanumba districts (Figure 1). It has a population of 126,213, with 62,881 males and 63,332 females [26]. The district has 250 communities, and it is found within the tropical climatic zone with Guinea savannah woodland vegetation. Ethnic groups found in the Kpandai district include Nawuris, Komkombas, Baasare and Kotokoli [26]. These two districts have some rivers and streams close to communities, which provide suitable breeding grounds for the vectors (blackflies) of *O. volvulus*.

The Nkwanta North and Kpandai districts started MDA with ivermectin in 1999. While treatment in the Kpandai District has not had any major breaks till date, treatment in Nkwanta North was halted in 2010 in some communities, including our study sites (due to ivermectin and other drug trials), and resumed in 2018 till date. The prevalence of onchocerciasis in these districts has seen a drastic decline over the years [27, 28]; however, there are still pockets of areas that continue to report infections with some communities having a microfilarial prevalence above 1% [27–29].

### 2.3. Study Design

This was a cross‐sectional study where participants with nodules were identified through community engagements with the support of the Ghana Health Service district health directorate. Participants were residents aged 18 years and above with palpable nodules who consented to be part of the study. Participants were selected by convenient sampling at the health centre of each community and palpable nodules were examined before selection. Questionnaires were administered to seek information about their demographics and treatment history. Additionally, treatment data of each community was obtained from the district health directorate. Sampling was carried out from November 2024 to February 2025.

### 2.4. Nodulectomy

Those who consented to have their nodules excised were initially skin snipped to determine infection. Nodules were then isolated by a trained surgeon at the health centre in the community using local anaesthesia. Isolated nodules were trimmed and placed in medium 199 and stored in liquid nitrogen for transportation to the laboratory at the Noguchi Memorial Institute for Medical Research (NMIMR) for further processing.

### 2.5. Laboratory Analysis (Nodule Digestion and Worm Isolation)

Frozen nodules were allowed to thaw. The nodules were further cleaned to remove all other extraneous tissues. The nodules were placed in a fresh 50 mL tube containing 5–10 mL of 0.5% collagenase A (Roche Diagnostics, Germany) in a sterile medium 199 solution supplemented with Earle’s salt, L‐glutamate (GIBCOTM, Life Technologies, Invitrogen Corporation, United States), sodium bicarbonate and 0.2 mg/mL Gentamicin sulphate. The nodules were then incubated in a bio‐shaker at 37°C and shaken at 150 rpm for 10–20 h to ensure complete digestion. The worms were isolated with the aid of an inverted microscope, a pair of forceps and a sterile plastic inoculating loop after the nodules were completely digested. Male and female adult worms that were found to be intertwined were gently separated to avoid fragmenting them. The worms were further rinsed individually in 0.9% phosphate buffered saline (PBS) in a petri dish and stored in absolute ethanol and in −20°C freezer until further processing.

### 2.6. Molecular Analysis (DNA Extraction)

Genomic DNA was extracted using the ZymoBIOMICS Pathogen DNA miniprep kit. A segment measuring about 1 cm length was cut from the tail end of each female adult worm (avoiding the uterus to prevent contamination) for the DNA extraction. Each segment was homogenised in 200 *μ*L of lysis buffer with a sterile grinding pestle. This was then followed for the entire DNA extraction procedure following the manufacturer’s protocol and the DNA eluted in 50 *μ*L of DNase/RNase‐Free water.

### 2.7. Genomic DNA Amplification and Digestion

Polymerase chain reaction (PCR) targeting the two genes (*P-gp* and *B-tub*) was carried out on the Genomic DNA samples using the TP600 PCR Thermal cycler (TaKaRa Bio Inc.). The standard PCR conditions were as follows: 95°C for 3 min, followed by 33 cycles of 95°C for 1 min, 45°C–55°C (based on the gene) for 45 s, 72°C for 1 min and a final extension of 72°C for 5 min. The target primer sequences are shown in Table 1. Following the PCR, restriction fragment length polymorphism (RFLP) assays were carried out on the PCR products, using *Dde*I and *Alu*I (Invitrogen Life Technologies) for *P-gp* and *B-tub* genes, respectively [23]. The PCR products were incubated with the respective restriction enzymes at 37°C for 2 h. The final digested products were run on a 2% agarose gel stained with 0.13 *μ*g/mL of Safeview for 1 h. Subsequent visualisation was done using a UV trans‐illuminator (BioDoC‐It Imaging System, Benchtop UV Transilluminator).

**Table 1:** Primer sequences and PCR conditions for amplification.

Primer name	Primer sequence	Annealing temp. (°C)	Band size (bp)
P‐glycoprotein‐like‐protein F	5 ^′^‐GATGGTTGAGGTTGAGGTTGAG‐3 ^′^	45	365
P‐glycoprotein‐like‐protein_R	5 ^′^‐GCCTATTACATGGAAGTGAG‐3 ^′^	45	365
Beta‐tubulin (isotype 1) F	5 ^′^‐GACACAGTTGGGGTAAAGGCAC‐3 ^′^	55	300
Beta‐tubulin (isotype 1) R	5 ^′^‐GTGTTGACCAGGGAATGCCTC‐3 ^′^	55	300

### 2.8. Sample Size Estimation

A conventional prospective sample‐size calculation was not performed because the study involved the recovery of adult *O. volvulus* worms from palpable onchocercomas in communities with a long history of ivermectin MDA. The availability of suitable parasites was therefore the primary limiting factor. Participants with palpable nodules who fulfilled the study eligibility criteria were recruited, and all nodules from which adult *O. volvulus* worms were successfully recovered were included in the molecular analysis. The study was designed primarily to characterise the occurrence and frequency of *P-gp* and *B-tub* polymorphisms in the available residual parasite population. Hence, comparisons between communities are interpreted as exploratory given the limited sample sizes.

### 2.9. Data Analysis

Data was entered and analysed using the Statistical Package for Social Sciences (SPSS, Version 25). The difference in frequency of the mutations between study sites was estimated using the chi‐square test. The association between the number of years of ivermectin treatment and the frequency of the mutations was estimated using regression analysis. The distribution of mutations across the endemic communities was mapped using Quantum Geographic Information System (QGIS) software, to provide a visual genetic landscape and pattern of the mutations.

## 3. Results

### 3.1. Demographic Information of Participants and Community Treatment History

A total of 86 participants who consented were involved in the nodulectomy procedure, but *O. volvulus* worms were successfully isolated from 33 participants whose data have been included in this study (for embryogram and genotyping). These formed individuals whose demographic information and treatment history have been presented in this study. The female participants were 63.6% while the males were 36.4% with average ages of 51 (21–73) years and 34 (20–55) years, respectively (*N* = 33). The participants indicated that they had lived in the community for more than 10 years, with about half living in the community from birth (Table 2). Majority of the participants also showed that they had been involved in ivermectin treatment for more than 10 times although the number varied from community to community (67%–100%). Additionally, about 88% of them indicated that they had been involved in the bi‐annual (twice a year) treatment (Table 2). Data also showed that there had been 21–29 rounds of ivermectin treatments in the communities before the commencement of this study (Table 2).

**Table 2:** Ivermectin treatment information of study participants.

Variable	Abunyanya 1 *N* (%)	Abunyanya 2 *N* (%)	Koni *N* (%)	Wiae *N* (%)	Overall *N* (%)
Duration lived in community					
< 1 year	0 (0.0)	0 (0.0)	0 (0.0)	0 (0.0)	0 (0.0)
1–5 years	0 (0.0)	0 (0.0)	0 (0.0)	0 (0.0)	0 (0.0)
6–10 years	0 (0.0)	0 (0.0)	0 (0.0)	0 (0.0)	0 (0.0)
11–17 years	5 (41.7)	6 (54.5)	4 (50.0)	0 (0.0)	15 (45.5)
From birth (≥18 years)	7 (58.3)	5 (45.5)	4 (50.0)	2 (100.0)	18 (54.5)

Frequency of IVM intake					
< 1 time	2 (16.7)	1 (9.1)	0 (0.0)	0 (0.0)	3 (9.1)
1–5 times	2 (16.7)	0 (0.0)	0 (0.0)	0 (0.0)	2 (6.1)
6–10 times	0 (0.0)	0 (0.0)	2 (25.0)	0 (0.0)	2 (6.1)
> 10 times	8 (66.7)	10 (90.9)	6 (75.0)	2 (100.0)	26 (78.8)

Overall rating of treatment frequency					
Once a year	0 (0.0)	0 (0.0)	0 (0.0)	0 (0.0)	0 (0.0)
Twice a year	9 (75.0)	10 (91.0)	8 (100.0)	2 (100.0)	29 (87.9)
Once every 2 years	0 (0.0)	0 (0.0)	0 (0.0)	0 (0.0)	0 (0.0)
Once every 5 years	0 (0.0)	0 (0.0)	0 (0.0)	0 (0.0)	0 (0.0)
No treatment for 10 years	3 (25.0)	1 (9.0)	0 (0.0)	0 (0.0)	4 (12.1)

Treatment frequency for the last 10 years					
Once	1 (8.3)	0 (0.0)	0 (0.0)	0 (0.0)	1 (3.0)
2–4 times	1 (8.3)	0 (0.0)	0 (0.0)	0 (0.0)	1 (3.0)
5–7 times	0 (0.0)	0 (0.0)	0 (0.0)	0 (0.0)	0 (0.0)
8–10 times	0 (0.0)	0 (0.0)	2 (25.0)	0 (0.0)	2 (6.1)
> 10 times	8 (66.7)	10 (91.0)	6 (75.0)	2 (100.0)	26 (78.8)
Never taken	2 (16.7)	1 (9.0)	0 (0.0)	0 (0.0)	3 (9.1)

No. of rounds of ivermectin treatment in the community					
Community treatment rounds	21	21	21	29	NA

*Note:* *N* = number of respondents to a particular question. Figures in parenthesis represent percentages of the respondents out of the total number of participants in each community. The total number of participants was 33. These comprised of sampling from each community as follows: Abunyaya 1 = 12; Abunyaya 2 = 11; Koni = 8; Wiae = 2.

Abbreviation: NA = not applicable.

### 3.2. Parasites Isolated From Participants

Overall, 36 nodules were obtained from the 33 participants, with the number of nodules per participant being 1.1. Out of these nodules, 45 adult female worms and 13 male worms were isolated, corresponding to an average of 1.3 and 0.4 female and male worms per nodule, respectively. About 4% of the female worms were identified with the morulae form, while 42% were identified with the stretched microfilariae within the uterus.

### 3.3. Treatment History of Patients in All Communities

The treatment of participants in the study areas was largely by MDA activities in the districts, which had gone on over the years. From the pre‐nodulectomy survey, the number of times participants indicated they had received ivermectin treatment varied from more than 10 years to none (never taken), as shown in Table 2. The majority (79%) of the participants had received more than 10 rounds of MDA, while 12% had received 1–10 rounds and 9% indicated never received treatment.

### 3.4. Worm Genetic Information Among Study Communities

The study focused on genotyping the 45 female worms isolated from the 33 participants (average of 1.4 worms per participant). These were analysed for the presence of wild‐type and mutant *B-tub* and *P-gp* genes. The highest number of worms were observed in Abunyanya 1 and Koni (with the highest ratio of worm/participant observed in Koni = 1.9 worm/participant and the least in Wiae = 1 worm/participant). For *B-tub*, 91.1% were identified as the mutant gene (ranged between 86.7% in Koni and 100% in Wiae) and 8.9% were wild type for *B-tub* while for *P-gp*, all worms were identified with a mutant gene for *P-gp* (Table 3).

**Table 3:** Distribution of wild type and mutant adult female worms in the study communities.

Community	Number of participants	No. of adult female worms analysed	Beta tubulin		P‐glycoprotein	
			Wild type *N* (%)	Mutant *N* (%)	Wild type *N* (%)	Mutant *N* (%)
Abunyanya 1	12	16	1 (6.3)	15 (93.7)	0 (0.0)	16 (100.0)
Abunyanya 2	11	12	1 (8.3)	11 (91.7)	0 (0.0)	12 (100.0)
Koni	8	15	2 (13.3)	13 (86.7)	0 (0.0)	15 (100.0)
Wiae	2	2	0 (0.0)	2 (100.0)	0 (0.0)	2 (100.0)
Total	33	45	4 (8.9)	41 (91.1)	0 (0.0)	45 (100.0)

*Note:* *N* = number of adult female worms. Figures in parenthesis represent percentages out of the total number of female worms collected in each community. Abunyaya 1, Abunyaya 2 and Koni = 8 had received 21 rounds of ivermectin treatment while Wiae had received 29 rounds of ivermectin treatment before the commencement of this study.

### 3.5. Association Between Community Rounds of Ivermectin Treatment and Frequency of Genetic Mutation of B‐tub and P‐gp

All the four communities had received either 21 or 29 rounds of ivermectin before commencement of the study. Even though the mutations were high and there had been many rounds of ivermectin treatment in the communities, there was no statistically significant association between the rounds of treatment and the genetic mutations for *B-tub* (Fisher^’^s Exact Test = 7.023; *p* = 0.621) and (Mann–Whitney *U* test = 57.000; Z = −0.554; *p* = 0.580) [Tables S1 and S2]. Similarly for *P-gp*, there was no statistically significant association observed between the rounds of ivermectin treatment and the mutations, since all were the mutant form (Mann–Whitney *U* test = 63.000; Z = 0.000; *p* = 1.000) [Table S2].

### 3.6. Effect of Ivermectin Treatment History of Individual Participant on the Frequency of B‐tub and P‐gp Genes Among the Adult Female Worms

At the individual participant level, we determined if there was any effect of individual participant’s ivermectin treatment frequency on the mutation of *B-tub* and *P-gp* genes among adult female worms. For the *B-tub* gene, the test showed no significant difference in mean ranks of frequency of mutation across the treatment groups (Kruskal–Wallis H = 2.606; df = 4; *p* = 0.626) [Table S3]. Similarly, for the *P-gp* gene, there was no significant difference in mean ranks of frequency of mutation (Kruskal–Wallis H = 0.000; df = 4; *p* = 0.953) [Table S3]. These results indicate that the number of ivermectin treatments of participants did not have a statistically significant effect on the frequency of mutation for both genes.

### 3.7. Geographic Distribution of Mutations Across the Endemic Communities

Figure 2 shows a visual display of the mutation frequencies of the two genes across the study communities. Geographically, there appears to be a difference in the distribution of the mutations for *B-tub* with Koni showing the least of the mutations and Wiae with the highest of the mutations and these two communities are distant in space (Figure 2a). This was not observed for *P-gp* since only the mutant forms were observed for the samples collected from all the communities (Figure 2b).

**Figure 2:**
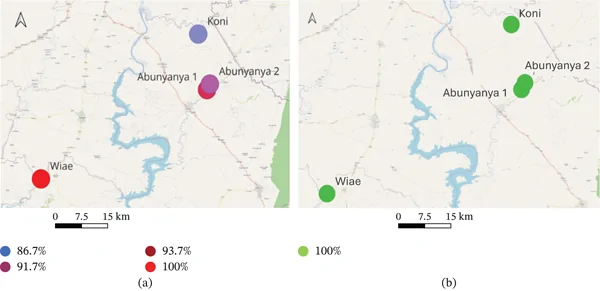
Distribution of genes across the four communities. Where (a) is the distribution of *B-tub* and (b) shows the distribution of *P-gp*. For *B-tub*, there were variations in the proportions of the mutant form among the four communities from Abunyanya 1, Abunyanya 2, Koni and Wiae showing 86.7%, 91.7%, 93.7% and 100%, respectively. On the other hand, all the adult female worms collected from all four communities had the *P-gp* mutant form.

## 4. Discussion

Onchocerciasis elimination strategy with MDA using ivermectin is likely to face some challenges due to reported suboptimal responses in parasite population [16, 30]. Even though this strategy has been effective in interrupting transmission [27, 28], especially with high coverage, the complete elimination of disease from endemic communities will involve the absence of the adult worm from endemic communities. Our study focused on the frequency of mutations in the *B-tub* and *P-gp* genes within adult female worms of *O. volvulus* in four onchocerciasis‐endemic communities in Ghana.

The presence of these adult worms in the participants we screened confirms the existence of onchocerciasis infection within these endemic communities. The stretched microfilariae observed in the uterus of about 40% of these adult female worms is an indication of reproductively active worms driving the residual transmission. This data supports previous studies in same communities suggesting residual transmission [16, 27]. The target towards elimination is a total absence of the adult worm and not only interruption of transmission; thus, studies on the adult worms are also important towards determining solutions to eliminate these parasites.

In genotyping the adult female worms, we focused on *B-tub* and *P-gp* genes because these two genes have been strongly associated with suboptimal response to ivermectin treatment in parasite population. We observed high frequencies of the mutation in these communities, and this is of great concern, with 87%–100% for *B-tub* and almost fixed for *P-gp. P-gp* is involved in the efflux pumping of drugs from the cytoplasm as an ABC transporter [31]. As far as treatment of onchocerciasis with ivermectin is concerned, it has been identified as one of the significant genes among others, where SNP can lead to suboptimal response in parasite population [23]. The uniformity of this mutation frequency can be attributed to the complete fixation of the mutated *P-gp* allele, which shows a strong selection pressure exerted by the prolonged exposure of the parasites to ivermectin by MDA activities. Considering the rounds (21–29 rounds) of ivermectin treatment our communities had received before the commencement of this study, and the active involvement of participants in treatments over the years (majority > 10 times treatment), it is not surprising that these genes are fixed or almost fixed in the parasite population. In such communities, the sole use of ivermectin towards elimination could be challenging and may require the use of other drugs such as moxidectin or doxycycline as well as other control strategies including vector control.

We did not find any statistically significant association between genetic variants and ivermectin treatment (at both community and individual levels) for both genes. This finding should, however, be interpreted cautiously because all study communities had been exposed to long‐term ivermectin treatment, having received at least 21 treatment rounds since 1999. Consequently, the study lacked ivermectin‐naïve communities that could provide a pre‐selection or untreated baseline for comparison. Previous investigations of ivermectin‐associated genetic selection in *O. volvulus* have used ivermectin‐naïve populations, pre‐treatment samples or untreated controls to establish baseline allele and genotype frequencies and subsequently evaluated treatment‐associated changes [32]. The absence of such a comparator in our study may therefore have limited our ability to distinguish treatment‐associated selection from pre‐existing genetic variation among the heavily treated parasite populations.

Mapping the frequencies of the genetic variants revealed apparent geographical differences in the distribution of *B-tub* mutations among the study communities. Koni had the lowest frequency of the mutations, whereas Wiae had the highest frequency, and these communities were geographically distant from each other. This pattern may suggest spatial heterogeneity in the distribution of *B-tub* variants among the *O. volvulus* populations. Geographic separation, restricted gene flow, genetic drift, local transmission dynamics and differences in historical drug‐selection pressure may all contribute to spatial variation in parasite genetic composition. However, other factors apart from geographical distance could have contributed to the genetic differentiation in *O. volvulus* populations [33] and therefore further studies may be needed to establish any spatial heterogeneity within these communities. In contrast, no comparable geographical variation was observed for the *P-gp* gene because only mutant forms were detected in samples from all study communities. Nonetheless, previous studies have shown that repeated ivermectin exposure can alter genetic diversity and allele frequencies at both *B-tub* and *P-gp* loci in *O. volvulus*, although the magnitude and spatial pattern of these changes may differ among parasite populations [32].

We acknowledge that our study had some limitations including the absence of an ivermectin‐naïve community of which these findings could have been compared with. However, in the current long‐term treatments, such naïve communities may not be in existence. We also acknowledge the effect of low number of samples obtained, especially from Wiae, the community with the longest treatment. Probably more samples could have presented some genetic diversity within the parasite population. This reflects the difficulty of obtaining parasites from endemic communities that have undergone prolonged ivermectin treatment, where palpable nodules and viable adult worms have become increasingly rare. The sampling of worms from a limited number of participants and nodules may reduce the effective number of independent observations. Therefore, our community‐level comparisons should be considered exploratory. Additionally, the technique of targeted gene approach identifying mutations using candidate genes instead of whole genome sequencing may have some limitations, since the treatment could have been associated with variation in other genes apart from *B-tub* and *P-gp*. Nonetheless, these data on the adult female worms are useful especially with *B-tub* and *P-gp* (strongly associated with ivermectin suboptimal response) in the parasite population. The study provides valuable molecular information on the residual adult *O. volvulus* population in a setting of prolonged ivermectin treatment and gives indication on the challenges programmes are likely to face using ivermectin MDA alone as the strategy towards elimination of onchocerciasis in Ghana and other similar settings.

## 5. Conclusion

Our data show a widespread occurrence of mutation for both *B-tub* and *P-gp* genes in *O. volvulus* adult female worms obtained from participants in all study communities, with an average of approximately 96%. Even though we observed these high frequencies, the study did not establish any statistically significant associations with community rounds of ivermectin treatment or individual treatment frequency, partly due to absence of ivermectin‐naïve community or prolonged treatments across these communities over the years. These findings will contribute to informing national and global onchocerciasis elimination strategies and provide a guide on whether to modify current MDAs, introduce other effective therapeutic interventions or have a more integrated approach with ivermectin MDA as far as the disease is concerned to meet the elimination timelines by 2030.

## Author Contributions

R.A. and M.O. drafted the manuscript. M.O., M.S.A., S.O., S.S.M.D. and A.A.N. performed field investigations. R.A., M.O., M.S.A., S.S.M.D. and A.A.N. conducted laboratory analysis. I.F. and J.H.N.O. analysed the data. M.C.E., D.A.B. and K.K.F. conceptualised and designed the study. M.C.E. and K.K.F. supervised the study. M.C.E., M.O., J.H.N.O., M.S.A., S.S.M.D., A.A.N., M.N.D., M.K.A., S.P.B. and K.K.F. reviewed the manuscript.

## Funding

This study was supported by WHO‐TDR, B80297.

## Disclosure

All the authors reviewed and approved the final manuscript.

## Ethics Statement

Ethical approval for the study was sought from the Ghana Health Service (GHS‐ERC:017/02/24). All participants were adults and gave a written consent before participating in the study.

## Consent

All authors gave their consent for the publication.

## Conflicts of Interest

The authors declare no conflicts of interest.

## Supporting Information

Additional supporting information can be found online in the Supporting Information section.

## Supporting information


**Supporting Information 1** Table S1: The cross tabulation of community rounds of ivermectin treatment and frequency of genetic mutation for beta‐tubulin (wildtype and mutant form).

**Supporting Information 2** Table S2: A Chi‐square test results between community rounds of ivermectin treatment and frequency of genetic mutation for beta‐tubulin.

**Supporting Information 3** Table S3: A cross tabulation of community rounds of ivermectin treatment and frequency of genetic mutation for p‐glycoprotein (wildtype and mutant form).

**Supporting Information 4** Table S4: A Mann–Whitney test results between rounds of ivermectin treatment and genetic frequencies of beta‐tubulin and p‐glycoprotein.

**Supporting Information 5** Table S5: A Kruskal–Wallis test of comparison of frequency of mutation of the gene across five different times of ivermectin treatment for individual participants.

## Data Availability

All study data is included in the article.
