# Supplementary material for: Frequency of Mutations Within P‐Glycoprotein and Beta‐Tubulin Associated With Ivermectin Treatment in Onchocerca volvulus Adult Female Worm Populations in Some Onchocerciasis Endemic Communities in Ghana
Source: J Parasitol Res. 2026 Oct 7;2026:3136302. doi: 10.1155/japr/3136302 (PMC13643362; doi:10.1155/japr/3136302)
Supplement: Supplementary file 1 [file JAPR-2026-3136302-s001.docx]

**Supplementary Table 1: Cross tabulation of community rounds of ivermectin treatment and frequency of genetic mutation for beta-tubulin**

|  |  |  |  | **B-TUB** | |  |
| --- | --- | --- | --- | --- | --- | --- |
|  |  |  |  | **Wild type** | **Mutated** | **Total** |
| Round of treatment | 21 rounds |  | Count | 4 | 39 | 43 |
|  |  |  | % within B-TUB | 100.0% | 95.1% | 95.6% |
|  | 29 rounds |  | Count | 0 | 2 | 2 |
|  |  |  | % within B-TUB | 0.0% | 4.9% | 4.4% |
| Total |  |  | Count | 4 | 41 | 45 |
|  |  |  | % within B-TUB | 100.0% | 100.0% | 100.0% |

A total of 45 sampled worms were analyzed for the presence of Wild Type and Mutated B-TUB genes. Among these, 4 worms (8.9%) were identified as having Wild Type B-TUB genes after treatment, all of which originated from worms in the 21-round treatment group. In contrast, 41 worms (91.1%) carried Mutated genes after treatment, with 39 (95.1%) from the 21-round treatment group and 2 (4.9%) from the 29-round treatment group. These findings indicate a high prevalence of B-TUB mutations, particularly in the population exposed to 21 rounds of treatment.
