# Supplementary material for: Frequency of Mutations Within P‐Glycoprotein and Beta‐Tubulin Associated With Ivermectin Treatment in Onchocerca volvulus Adult Female Worm Populations in Some Onchocerciasis Endemic Communities in Ghana
Source: J Parasitol Res. 2026 Oct 7;2026:3136302. doi: 10.1155/japr/3136302 (PMC13643362; doi:10.1155/japr/3136302)
Supplement: Supplementary file 2 [file JAPR-2026-3136302-s002.docx]

**Supplementary Table 2: Chi-square test between community rounds of ivermectin treatment and frequency of genetic mutation for beta-tubulin**

|  | **Value** | **df** | **Asymptotic Significance (2-sided)** | **Exact Sig. (2-sided)** | **Exact Sig. (1-sided)** |
| --- | --- | --- | --- | --- | --- |
| Pearson Chi-Square | .314^a^ | 1 | .575 | 0.602 | .751 |
| Continuity Correction^b^ | .000 | 1 | 1.000 |  |  |
| Likelihood Ratio | .579 | 1 | .447 | .996 | .751 |
| Fisher's Exact Test | 7.023 |  |  | .621 | .751 |
| Linear-by-Linear Association | .307^c^ | 1 | .580 | .981 | .751 |
| N of Valid Cases | 45 |  |  |  |  |

The results indicated a Fisher’s Exact Test value of 7.023 with a p-value of 0.621, which is above the conventional threshold of 0.05, demonstrating that the association between rounds of treatment and frequency of mutation of the B-TUB gene is not statistically significant. These findings suggest that the distribution of Wild Type and Mutated B-TUB genes does not differ significantly between the 21-round and 29-round treatment populations.
