# Supplementary material for: Frequency of Mutations Within P‐Glycoprotein and Beta‐Tubulin Associated With Ivermectin Treatment in Onchocerca volvulus Adult Female Worm Populations in Some Onchocerciasis Endemic Communities in Ghana
Source: J Parasitol Res. 2026 Oct 7;2026:3136302. doi: 10.1155/japr/3136302 (PMC13643362; doi:10.1155/japr/3136302)
Supplement: Supplementary file 3 [file JAPR-2026-3136302-s003.docx]

**Supplementary Table 3: Cross tabulation of community rounds of ivermectin treatment and frequency of genetic mutation for p-glycoprotein**

|  |  |  | **P-GP** |  |
| --- | --- | --- | --- | --- |
|  |  |  | **Mutated** | **Total** |
| Round of treatment | 21 rounds | Count | 43 | 43 |
|  |  | % within P-GP | 95.6% | 95.6% |
|  | 29 rounds | Count | 2 | 2 |
|  |  | % within P-GP | 4.4% | 4.4% |
| Total |  | Count | 45 | 45 |
|  |  | % within P-GP | 100.0% | 100.0% |

A total of 45 sampled worms were analyzed for the presence of Wild Type and Mutated P-GP genes. All (45) of the worm were identified to have carried Mutated genes after treatment, with 43 (95.6%) from the 21-round treatment communities and 2 (4.4%) from the 29-round treatment community. These findings indicate a high prevalence of P-GP mutations.
