# Supplementary material for: Frequency of Mutations Within P‐Glycoprotein and Beta‐Tubulin Associated With Ivermectin Treatment in Onchocerca volvulus Adult Female Worm Populations in Some Onchocerciasis Endemic Communities in Ghana
Source: J Parasitol Res. 2026 Oct 7;2026:3136302. doi: 10.1155/japr/3136302 (PMC13643362; doi:10.1155/japr/3136302)
Supplement: Supplementary file 4 [file JAPR-2026-3136302-s004.docx]

**Supplementary Table 4: Mann Whitney test between rounds of ivermectin treatment and genetic frequencies of B-tub and P-gp**

|  | **Round of treatment** | **N** | **Mean Rank** | **Sum of Ranks** |
| --- | --- | --- | --- | --- |
| B-TUB | 21 rounds | 43 | 22.86 | 982.98 |
|  | 29 rounds | 2 | 25.00 | 50.00 |
|  | Total | 45 |  |  |
| P-GP | 21 rounds | 43 | 23.00 | 989.00 |
|  | 29 rounds | 2 | 23.00 | 46.00 |
|  | Total | 45 |  |  |

For the B-TUB gene, the 21-round treatment group had a mean rank of 22.86, while the 29-round treatment group had a mean rank of 25.00 indicating a slide difference in the frequency of mutation between the two groups. In contrast, for the P-GP gene, both the 21- and 29-round treatment groups had identical mean ranks of 23.00, indicating no apparent difference between the groups.

A Mann-Whitney U test was conducted to compare the frequency of mutation of B-TUB and P-GP genes between the 21-round and 29-round treatment communities. For the B-TUB gene, the 21-round group had a mean rank of 22.86, and the 29-round group had a mean rank of 25.00. The test indicated no significant difference between the groups, U = 57.000, Z = -0.554, p = 0.580 (Exact Sig. = 0.814). For the P-GP gene, both groups had a mean rank of 23.00, and no significant difference was observed, U = 63.000, Z = 0.000, p = 1.000 (Exact Sig. = 0.994). These results suggest that the rounds of treatment had no significant effect on the frequency of mutation for both genes.
