# Supplementary material for: Frequency of Mutations Within P‐Glycoprotein and Beta‐Tubulin Associated With Ivermectin Treatment in Onchocerca volvulus Adult Female Worm Populations in Some Onchocerciasis Endemic Communities in Ghana
Source: J Parasitol Res. 2026 Oct 7;2026:3136302. doi: 10.1155/japr/3136302 (PMC13643362; doi:10.1155/japr/3136302)
Supplement: Supplementary file 5 [file JAPR-2026-3136302-s005.docx]

**Supplementary Table 5: Kruskal Wallis test of comparison of frequency of mutation of the gene across five different times of ivermectin treatment**

|  | **Number of times ivermectin was taken** | **N** | **Mean Rank** |
| --- | --- | --- | --- |
| B-TUB | Once | 6 | 25.00 |
|  | 2-4 times | 4 | 19.38 |
|  | 8-10 times | 4 | 25.00 |
|  | > 10 times | 28 | 22.59 |
|  | Never taken | 3 | 25.00 |
|  | Total | 45 |  |
| P-GP | Once | 6 | 23.00 |
|  | 2-4 times | 4 | 23.00 |
|  | 8-10 times | 4 | 23.00 |
|  | > 10 times | 28 | 23.00 |
|  | Never taken | 3 | 23.00 |
|  | Total | 45 |  |

The distribution of mean ranks for frequency of mutation of B-TUB gene varied according to the number of times ivermectin was taken. Worms treated with ivermectin once, 8–10 times or never had the highest mean ranks (25.00), whereas worms treated with ivermectin 2–4 times had the lowest mean rank (19.38). For worms treated with ivermectin >10 times, the mean rank was 22.59. In contrast, for the P-GP gene, all groups had identical mean ranks of 23.00, indicating no apparent difference based on ivermectin treatment frequency. These results suggest that the number of ivermectin treatments may slightly influence mutation of B-TUB gene but has no effect on mutation of P-GP gene.

A Kruskal-Walli’s test was conducted to examine the effect of ivermectin treatment frequency on the frequency of mutation of B-TUB and P-GP genes among worms. For the B-TUB gene, the test showed no significant difference in mean ranks of frequency of mutation across the five groups, H(4) = 2.606, p = 0.626. Similarly, for the P-GP gene, there was no significant difference in mean ranks of frequency of mutation, H(4) = 0.000, p = 0.953. These results indicate that the number of ivermectin treatments does not have a statistically significant effect on the frequency of mutation of either genes.
